# Genome sequence of the pink–pigmented marine bacterium *Loktanella hongkongensis* type strain (UST950701–009P^T^), a representative of the *Roseobacter* group

**DOI:** 10.1186/s40793-015-0050-9

**Published:** 2015-08-11

**Authors:** Stanley CK Lau, Thomas Riedel, Anne Fiebig, James Han, Marcel Huntemann, Jörn Petersen, Natalia N. Ivanova, Victor Markowitz, Tanja Woyke, Markus Göker, Nikos C. Kyrpides, Hans-Peter Klenk, Pei-Yuan Qian

**Affiliations:** Division of Life Science, The Hong Kong University of Science and Technology, Clear Water Bay, Hongkong, People’s Republic of China; Leibniz Institute DSMZ – German Collection of Microorganisms and Cell Cultures, Braunschweig, Germany; Helmholtz Centre for Infection Research, Braunschweig, Germany; DOE Joint Genome Institute, Walnut Creek, CA USA; Biological Data Management and Technology Center, Lawrence Berkeley National Laboratory, Berkeley, CA USA

**Keywords:** Biofilms, Marine, *Roseobacter* group, *Rhodobacteraceae*, *Alphaproteobacteria*, Plasmids

## Abstract

*Loktanella hongkongensis* UST950701-009P^T^ is a Gram-negative, non-motile and rod-shaped bacterium isolated from a marine biofilm in the subtropical seawater of Hong Kong. When growing as a monospecies biofilm on polystyrene surfaces, this bacterium is able to induce larval settlement and metamorphosis of a ubiquitous polychaete tubeworm *Hydroides elegans*. The inductive cues are low-molecular weight compounds bound to the exopolymeric matrix of the bacterial cells. In the present study we describe the features of *L. hongkongensis* strain DSM 17492^T^ together with its genome sequence and annotation and novel aspects of its phenotype. The 3,198,444 bp long genome sequence encodes 3104 protein-coding genes and 57 RNA genes. The two unambiguously identified extrachromosomal replicons contain replication modules of the RepB and the *Rhodobacteraceae*-specific DnaA-like type, respectively.

## Introduction

*Loktanella hongkongensis* UST950701-00P^T^ (= DSM 17492^T^ = NRRL B-41039^T^ = JCM 12479^T^) was isolated from a biofilm grown naturally on a glass coupon that had been submerged in the coastal seawater of Hong Kong for 7 days in July 1995 [1]. In the marine environment, bacteria in biofilms mediate the settlement and metamorphosis of the planktonic larvae of many benthic invertebrates. The cells of UST950701-00P^T^, when attached as a biofilm, were able to induce settlement and metamorphosis of the polychaete *Hydroides elegans* [2]. The chemical cues mediating the larval response were found to be low-molecular weight compounds associated with the exopolymeric matrix of the bacterial cells [3–5].

In this study we analyzed the genome sequence of *L. hongkongensis* DSM 17492^T^. We present a description of the genome sequencing, an annotation and a summary classification together with a set of features for strain, including novel aspects of its phenotype.

## Organism information

### Classification and features

**Fig. 1:**
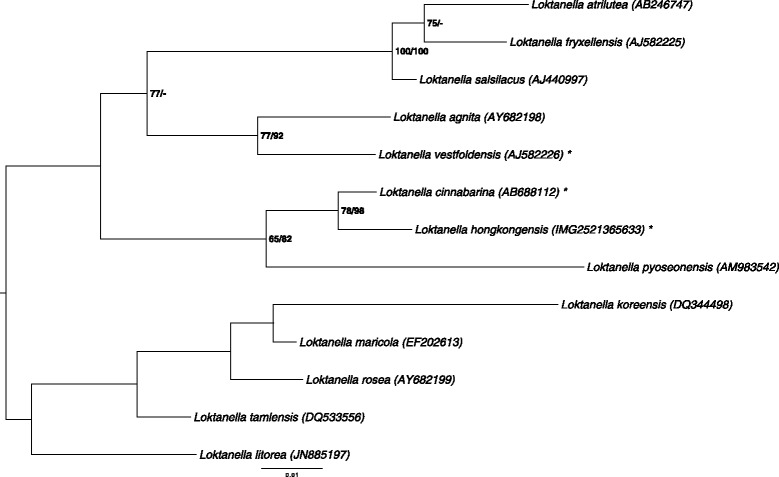
Phylogenetic tree highlighting the position of *L. hongkongensis* relative to the type strains of the other species within the genus *Loktanella* [6, 13]. The tree was inferred from 1353 aligned characters of the 16S rRNA gene sequence under the maximum likelihood (ML) criterion as previously described [14]. Rooting was done initially using the midpoint method and then checked for its agreement with the current classification (Table 1). The branches are scaled in terms of the expected number of substitutions per site. Numbers adjacent to the branches are support values from 350 ML bootstrap replicates (left) and from 1000 maximum-parsimony bootstrap replicates (right) if larger than 60 % [6]. Lineages with type strain genome sequencing projects registered in GOLD [7] are labeled with one asterisk, those also listed as ‘Complete and Published’ with two asterisks

Figure 1 shows the phylogenetic neighborhood of L. hongkongensis DSM 17492^T^ in a 16S rRNA gene based tree. The sequence of the single 16S rRNA gene copy in the genome does not differ from the previously published 16S rRNA gene sequence (AY600300).

The single genomic 16S rRNA gene sequence of *L. hong-kongensis* DSM 17492^T^ was compared with the Greengenes database for determining the weighted relative frequencies of taxa and (truncated) keywords as previously described [6]. The most frequently occurring genera were *Loktanella* (46.2 %), *Ketogulonicigenium* (14.9 %), *Methylarcula* (10.3 %), *Silicibacter* (10.0 %) and *Ruegeria* (8.5 %) (65 hits in total). Regarding the five hits to sequences from representatives of the species, the average identity within high-scoring segment pairs was 99.6 %, whereas the average coverage by HSPs was 98.0 %. Regarding the 13 hits to sequences from other representatives of the genus, the average identity within HSPs was 95.6 %, whereas the average coverage by HSPs was 97.6 %. Among all other species, the one yielding the highest score was *Loktanella vestfoldensis* (NR_029021), which corresponded to an identity of 95.8 % and a HSP coverage of 99.4 %. (Note that the Greengenes database uses the INSDC (= EMBL/NCBI/DDBJ) annotation, which is not an authoritative source for nomenclature or classification). The highest-scoring environmental sequence was FJ869048 (Greengenes short name ‘*Roseobacter* isolates Chesapeake Bay water 2 m depth isolate CB1079Rhodobacterales str. CB1079’), which showed an identity of 99.2 % and an HSP coverage of 99.9 %. The most frequently occurring keywords within the labels of all environmental samples which yielded hits were ‘lake’ (8.6 %), ‘tin’ (7.1 %), ‘qinghai’ (6.4 %), ‘microbi’ (3.2 %) and ‘sea’ (3.1 %) (185 hits in total). The most frequently occurring keywords within the labels of those environmental samples which yielded hits of a higher score than the highest scoring species were ‘sea’ (15.4 %), ‘water’ (7.7 %), ‘bloom, chl, concentr, contrast, diatom, dure, filter, non-bloom, spring, station, success, surfac, yel’ (5.1 %) and ‘bai, chesapeak, depth, roseobact’ (2.6 %) (3 hits in total). These keywords fit well to the isolation site of strain UST950107-009P^T^.

**Fig. 2:**
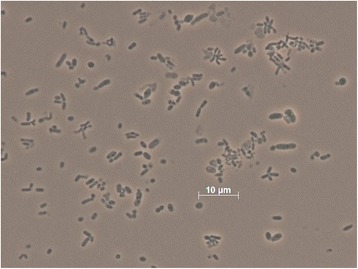
Phase-contrast micrograph of strain *L. hongkongensis* DSM 17492^T^

*L. hongkongensis* UST950107-009P^T^ is Gram-negative and non-spore forming (Table 1). Cells are short rods and non-motile (Fig. 2). When grown on Marine Agar 2216 (Difco) at 30 ˚C in the absence of light, colonies are pink in color, convex with entire margin, and have smooth and shiny surface; brown diffusible pigment is produced. However, whitish colonies would emerge from every culture upon aging (3 days or beyond). The colonies of the white morphovar, with otherwise identical morphological properties, can be maintained as separate cultures (UST950701-009 W) without turning pink. *L. hongkongensis* UST950107-009P^T^ cannot grow on nutrient agar or trypticase-soy agar (both from Oxoid).

The growth of *L. hongkongensis* UST950701-009P^T^ is strictly aerobic and requires at least 2 % NaCl (up to 14 %). The ranges of temperature and pH where its growth can occur are 8–44 ˚C and 5.0–10.0, respectively. *L. hongkongensis* UST950107-009P^T^ can utilize a wide range of mono-, di-, tri- and polysaccharides, and sugar alcohols. Citrate is not utilized. Catalase, oxidase and beta-galactosidase activities are positive whereas arginine dihydrolase, lysine decarboxylase, ornithine decarboxylase, urease, tryptophane deaminase and gelatinase are negative. *L. hongkongensis* UST950701-009P^T^ does not produce bacteriochlorophyll *a*, indole, acetoin or H_2_S. It cannot hydrolysis casein or tween 80. Streptomycin, penicillin, chloramphenicol, amplicilin and tetracycline can inhibit the growth of *L. hongkongensis* UST950107-009P^T^ but kanamycin cannot (all data from [1]).

The utilization of carbon compounds by *L. hongkongensis* DSM 17492^T^ grown at 28 °C was also determined for this study using Generation-III microplates in an OmniLog phenotyping device (BIOLOG Inc., Hayward, CA, USA). The microplates were inoculated at 28 °C with dye IF-A and a cell suspension at a cell density of 95–96 % turbidity. Further additives were vitamin, micronutrient and sea-salt solutions [14]. The plates were sealed with parafilm to avoid a loss of fluid. The exported measurement data were further analyzed with the opm package for R [15, 16], using its functionality for statistically estimating parameters from the respiration curves such as the maximum height, and automatically translating these values into negative, ambiguous, and positive reactions. The reactions were recorded in three individual biological replicates. Positive results were received for the following substrates: positive control, pH 6, 1 % NaCl, 4 % NaCl, 8 % NaCl, D-galactose, 3-O-methyl-D-glucose, D-fucose, L-fucose, L-rhamnose, inosine, 1 % sodium lactate, myo-inositol, rifamycin SV, L-aspartic acid, L-glutamic acid, L-histidine, L-serine, D-glucuronic acid, glucuronamide, quinic acid, L-lactic acid, citric acid, *α*-keto-glutaric acid, D-malic acid, L-malic acid, nalidixic acid, acetic acid and sodium formate.

According to Generation-III plates the strain is negative for dextrin, D-maltose, D-trehalose, D-cellobiose, *β*-gentiobiose, sucrose, D-turanose, stachyose, pH 5, D-raffinose, *α*-D-lactose, D-melibiose, *β*-methyl-D-galactoside, D-salicin, *N*-acetyl-D-glucosamine, *N*-acetyl-β-D-mannosamine, *N*-acetyl-D-galactosamine, *N*-acetyl-neuraminic acid, D-glucose, D-mannose, D-fructose, fusidic acid, D-serine, D-sorbitol, D-mannitol, D-arabitol, glycerol, D-glucose-6-phosphate, D-fructose-6-phosphate, D-aspartic acid, D-serine, troleandomycin, minocycline, gelatin, glycyl-L-proline, L-alanine, L-arginine, L-pyroglutamic acid, lincomycin, guanidine hydrochloride, niaproof, pectin, D-galacturonic acid, L-galactonic acid-γ-lactone, D-gluconic acid, mucic acid, D-saccharic acid, vancomycin, tetrazolium violet, tetrazolium blue, *p*-hydroxyphenylacetic acid, methyl pyruvate, D-lactic acid methyl ester, bromo-succinic acid, lithium chloride, potassium tellurite, tween 40, *γ*-amino-n-butyric acid, *α*-hydroxy-butyric acid, *β*-hydroxybutyric acid, *α*-keto-butyric acid, acetoacetic acid, propionic acid, aztreonam, butyric acid and sodium bromate and the negative control.

The phenotype of the strain was described as well as the assimilation of a wide range of sugars was tested by Lau et al. [1] with the API50CH system, which is based on the detection of biochemical reactions. Using the API50CH system positive reactions were found for more than 20 carbon sources. None of these results could be confirmed by the OmniLog measurement. *L. hongkongensis* was positive for only five sugars, as well as for a number of carboxylic acids (e.g. malate and citrate) and amino acids. This observation agrees with the finding of Van Trappen et al. [6], who determined the phenotype of three Loktanella strains using API20NE, except for the difference that no positive reaction was found for the carbon sources given in [6]. Positive reactions found in the OmniLog measurements but not in growth experiments might be due to the higher sensitivity of the former [17].

#### Chemotaxonomy

The predominant fatty acids of *L. hongkongensis* UST950107-009P^T^ are C_18:1 Ω7C_ (84.5 %), C_16:0_ (5.8 %), C_18:0_ (3.5 %), C_10:0 3-OH_ (2.0 %) and C_12:0 3-OH_ (1.9 %), making up to 97.7 % of the total [1]. The remaining fatty acids are C_12:1 3-OH_, C17:O, C_18:1 Ω7C 11-methyl_, summed feature 3 (comprising C_16:1 Ω7c_ and C_15 iso 2-OH_), and an unknown peak with an expected chain length equivalent to 11.799.

## Genome sequencing and annotation

### Genome project history

The genome was sequenced within the project “Ecology, Physiology and Molecular Biology of the *Roseobacter* clade: Towards a Systems Biology Understanding of a Globally Important Clade of Marine Bacteria”. The strain was chosen for genome sequencing according to the *Genomic Encyclopedia of* Bacteria *and*Archaea criteria [29]. For the same reason it was previously also chosen as part of the “Genomic Encyclopedia of Type Strains, Phase I: the one thousand microbial genomes project” [51, 52], a follow-up of the GEBA project [30], which aims at increasing the sequencing coverage of key reference microbial genomes. Two draft sequences were produced independently from the same source of DNA and finally joined. According project information can found in the Genomes OnLine Database [31]. The Whole Genome Shotgun sequence is deposited in Genbank and the Integrated Microbial Genomes database (IMG) [32]. A summary of the project information is shown in Table 2.

**Table 1:** Classification and general features of *L. hongkongensis* UST950701-009P^T^ in accordance with the MIGS recommendations [18] published by the Genome Standards Consortium [19]

MIGS ID	Property	Term	Evidence code
	Classification	Domain *Bacteria*	TAS [20]
		Phylum *Proteobacteria*	TAS [21]
		Class *Alphaproteobacteria*	TAS [22, 23]
		Order *Rhodobacterales*	TAS [23, 24]
		Family *Rhodobacteraceae*	TAS [25]
		Genus *Loktanella*	TAS [6, 8–12, 13, 26]
		Species *Loktanella hongkongensis*	TAS [1]
		Strain UST950701-009P^T^	TAS [1]
	Gram stain	Negative	TAS [1]
	Cell shape	Short rods	TAS [1]
	Motility	Non-motile	TAS [1]
	Sporulation	Non-sporulating	TAS [1]
	Temperature range	8–44 °C	TAS [1]
	Optimum temperature	25–30 °C	NAS
MIGS-6.3	Salinity	2–14 %	TAS [1]
	pH range; Optimum	5.0–10.0; not determined	TAS [1]
MIGS-22	Oxygen requirement	Strictly aerobic	TAS [1]
	Carbon source	Sugar alcohols and polysaccharides	TAS [1]
	Energy metabolism	Chemoorganotrophy	TAS [1]
MIGS-6	Habitat	Marine biofilm	TAS [1]
MIGS-15	Biotic relationship	Free-living	NAS
MIGS-14	Pathogenicity	Not reported	
	Biosafety level	1	TAS [27]
MIGS-23.1	Isolation	Marine biofilm	TAS [1]
MIGS-4	Geographic location	Hong Kong	TAS [1]
MIGS-5	Sample collection	July 1995	NAS
MIGS-4.1	Latitude	22°20′16.28″ N	NAS
MIGS-4.2	Longitude	114°16′7.81″ E	NAS
MIGS-4.3	Depth	1 m during low tide	NAS
MIGS-4.4	Altitude	Not applicable	

Evidence codes - TAS: Traceable Author Statement (i.e., a direct report exists in the literature); NAS: Non-traceable Author Statement (i.e., not directly observed for the living, isolated sample, but based on a generally accepted property for the species, or anecdotal evidence). Evidence codes are from of the Gene Ontology project [28]

**Table 2:** Genome sequencing project information

MIGS ID	Property	Term
MIGS-31	Finishing quality	Non-contiguous finished
MIGS-28	Libraries used	Two genomic libraries: one Illumina PE library (500 bp insert size), one 454 PE library (3 kb insert size)
MIGS-29	Sequencing platforms	Illumina GA IIx, Illumina MiSeq, 454 GS-FLX + Titanium
MIGS-31.2	Fold coverage	132 ×
MIGS-30	Assemblers	Velvet version 1.1.36, Newbler version 2.3, Consed 20.0
MIGS-32	Gene calling method	Prodigal 1.4
	Genbank ID	APGJ00000000
	Genbank date of release	March 29, 2014
	GOLD ID	Gi22711
	BIOPROJECT	183668
MIGS-13	Source material identifier	DSM 17492^T^
	Project relevance	Tree of Life, environmental

### Growth conditions and genomic DNA preparation

A culture of strain DSM 17492^T^ was grown aerobically in DSMZ medium 514 [33] at 28 °C. Genomic DNA was isolated using Jetflex Genomic DNA Purification Kit (GENOMED 600100) following the standard protocol provided by the manufacturer but modified by an incubation time of 60 min, incubation on ice over night on a shaker, the use of additional 50 μl proteinase K, and the addition of 100 μl protein precipitation buffer. DNA is available from the DSMZ through the DNA Network [34].

### Genome sequencing and assembly

The genome was sequenced using a combination of two libraries (Table 2). Illumina sequencing was performed on a GA IIx platform with 150 cycles. The paired-end library contained inserts of an average of 500 bp in length. The first run on Illumina GAII platform delivered 1.0 million reads. A second Illumina run was performed on a Miseq platform to gain a higher sequencing depth. To achieve longer reads, the library was sequenced in one direction for 300 cycles, providing another 2.1 million reads. After error correction and clipping by fastq-mcf [35] and quake [36], the data was assembled using velvet [37]. A total of 2,403,257 reads with a mean length of 126 bp passed the filter step and were assembled in 54 contigs. To gain information on the contig arrangement an additional 454 run was performed. The paired-end jumping library of 3 kb insert size was sequenced on a 1/8 lane. Pyrosequencing resulted in 158,608 reads with an average length of 337 bp. A total of 41 scaffolds was obtained from Newbler assembler (Roche Diagnostics).

Both draft assemblies (Illumina and 454 sequences) were fractionated into artificial Sanger reads of 1000 nt in length plus 75 bp overlap on each site. These artificial reads served as an input for the phred/phrap/consed package [38]. By manual editing the number of contigs was reduced to 13. Using minimus2 [39], the resulting sequence was mapped to an existing permanent draft version of the genome published on IMG-ER by the DOE *Joint Genome Institute*, which was sequenced as described earlier [53]. The source DNA of both samples was obtained from the same origin DSM 17492^T^. The combined sequences provided a 132 × coverage of the genome.

### Genome annotation

Genes were identified using Prodigal [40] as part of the JGI genome annotation pipeline. The predicted CDSs were translated and used to search the National Center for Biotechnology Information nonredundant database, UniProt, TIGR-Fam, Pfam, PRIAM, KEGG, COG, and InterPro databases. Identification of RNA genes were carried out by using HMMER 3.0rc1 [41] (rRNAs) and tRNAscan-SE 1.23 [42] (tRNAs). Other non-coding genes were predicted using INFERNAL 1.0.2 [43] Additional gene prediction analysis and functional annotation was performed within the Integrated Microbial Genomes - Expert Review platform [44] CRISPR elements were detected using CRT [45] and PILER-CR [46].

## Genome properties

**Fig. 3:**
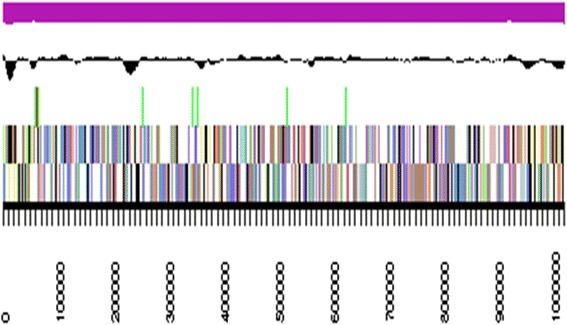
Graphical map of the largest scaffold. From bottom to the top: Genes on forward strand (colored by COG categories), Genes on reverse strand (colored by COG categories), RNA genes (tRNAs green, rRNAs red, other RNAs black), GC content (black), GC skew (purple/olive)

The genome statistics are provided in Table 3 and Fig. 3. The genome of strain DSM 17492^T^ has a total length of 3,198,444 bp and a G + C content of 68.3 %. Of the 3161 genes predicted, 3104 were identified protein-coding genes, and 57 RNAs. The majority of the protein-coding genes were assigned a putative function (83.9 %) while the remaining ones were annotated as hypothetical proteins. The distribution of genes into COGs functional categories is presented in Table 4.

**Table 3:** Genome statistics^a^

Attribute	Value	% of Total
Genome size (bp)	3,198,444	100.00
DNA coding region (bp)	2,899,639	90.66
DNA scaffolds	9	
Extrachromosomal elements	2	
Total genes	3161	100.00
RNA genes	57	1.80
rRNA operons	2	
tRNA genes	44	1.39
Protein-coding genes	3104	98.20
Genes with function prediction (proteins)	2652	83.90
Genes in paralog clusters	2546	80.54
Genes assigned to COGs	2566	81.18
Genes assigned Pfam domains	2697	85,32
Genes with signal peptides	291	9.21
Genes with transmembrane helices	704	22.27
CRISPR repeats	0	

^a^The annotation shown in IMG [32] is subject to regular updates; the numbers presented here might deviate from later versions of the genome

**Table 4:** Number of genes associated with the general COG functional categories

Code	Value	% age	Description
J	172	6.2	Translation, ribosomal structure and biogenesis
A	0	0.0	RNA processing and modification
K	167	6.0	Transcription
L	127	4.5	Replication, recombination and repair
B	2	0.1	Chromatin structure and dynamics
D	30	1.1	Cell cycle control, cell division, chromosome partitioning
Y	0	0.0	Nuclear structure
V	28	1.0	Defense mechanisms
T	103	3.7	Signal transduction mechanisms
M	179	6.4	Cell wall/membrane/envelope biogenesis
N	43	1.5	Cell motility
Z	0	0.0	Cytoskeleton
W	0	0.0	Extracellular structures
U	49	1.8	Intracellular trafficking and secretion, and vesicular transport
O	112	4.0	Posttranslational modification, protein turnover, chaperones
C	178	6.4	Energy production and conversion
G	185	6.6	Carbohydrate transport and metabolism
E	273	9.8	Amino acid transport and metabolism
F	75	2.7	Nucleotide transport and metabolism
H	125	4.5	Coenzyme transport and metabolism
I	108	3.9	Lipid transport and metabolism
P	141	5.0	Inorganic ion transport and metabolism
Q	77	2.8	Secondary metabolites biosynthesis, transport and catabolism
R	339	12.1	General function prediction only
S	282	10.1	Function unknown
-	595	18.8	Not in COGs

## Insights from the genome sequence

Genome sequencing of *L. hongkongensis* DSM 17492^T^ reveals the presence of two plasmids with sizes of about 85 kb and 103 kb (Table 5). These plasmids contain characteristic replication modules of the RepB and DnaA-like type comprising a replicase as well as the *parAB* partitioning operon. The respective replicases that mediate the initiation of replication are designated according to the established plasmid classification scheme [47]. The different numbering of the replicases (RepB-I, DnaA-like I) corresponds to specific plasmid compatibility groups that are required for a stable coexistence of the replicons within the same cell. Type-IV secretion systems for conjugative plasmid transfer [48, 49] and postsegregational killing systems, consisting of a typical operon with two small genes encoding a stable toxin and an unstable antitoxin [50], are missing on both plasmids. The presence of a RepA-I plasmid replicase (lokhon_02202) in close proximity to a complete rRNA operon on the chromosomal 1,0 MB contig 684.8 is conspicuous. The *parAB* partitioning operon is located 15 genes downstream of *repA-I* indicating that the replication module has been subjected to several recombination events with the chromosome and is probably not functional any more. However, genome finishing would be required to document the presence of a single chromosomal replicon in *L. hongkongensis* DSM 17492^T^.

**Table 5:** General genomic features of the chromosome and extrachromosomal replicons from *L. hongkongensis* strain DSM 17492^T^

Replicon	Contig	Replicase	Length (bp)	GC (%)	Topology	No. genes^b^
Chromosome^c^	644.4	DnaA	531,696	69	Linear^a^	540
Chromosome^c^	684.8	RepA-I	1,020,876	67	Linear^a^	984
Plasmid 1	47.0	RepB-I	85,337	70	Linear^a^	85
Plasmid 2	51.0	DnaA-like I	103,367	69	Linear^a^	87

^a^circularity not experimentally validated

^b^deduced from automatic annotation

^c^contigs representing the chromosome

## Conclusion

The marine *Roseobacter* group is widely distributed in the marine environment. In this study we analyzed the genome sequence of *L. hongkongensis* UST950701-009P^T^, which was isolated from a marine biofilm, and summarized known and newly revealed aspects of its phenotype. Genome analysis of this type strain demonstrated at least two extrachromosomal elements with replication systems specific or at least characteristic for the family *Rhodobacteraceae*.

## Abbreviations

DOE: Department of Energy
GEBA: Genomic encyclopedia of *Bacteria* and *Archaea*
HSP: High-scoring segment pair
IMG: Integrated microbial genomes
JGI: Joint Genome Institute
KMG: 1000 microbial genomes
WGS: Whole genome shotgun
